# Steve Oroszlan: A Personal Perspective

**DOI:** 10.3390/v13040622

**Published:** 2021-04-05

**Authors:** Raymond Gilden

**Affiliations:** 5802 Meadow Drive, Frederick, MD 21702, USA; rgilden1935@gmail.com

**Keywords:** retroviruses, HIV, viral proteins, virus maturation, viral immunology

## Abstract

My memories of Steve go back over 50 years. While precise dates are no longer in my memory bank, circumstances and emotions remain alive and easy to recall. These memories tell the story of a remarkable human being, a true practitioner of his craft always, faithful to the basic principles of scientific pursuit, with integrity, honesty, and enthusiasm well beyond the norm. We had a professional symbiotic relationship that lasted over 20 years, resulting in over 50 publications in scientific journals and meeting abstracts. During that time, our fortunes rose in tandem, and when it was time to go our separate ways, he was more than ready to flourish on his own. Our personal friendship remained constant, and we enjoyed sharing meals and stories with family and friends over the years. In retrospect, I take pride in having played a role in a portion of his remarkable scientific journey. A few key anecdotes will illustrate some aspects of this summary. By way of a disclaimer, this is not a comprehensive review of the vast field of viral oncology and the selection of references is intentionally narrow. No slight is intended to the many outstanding investigators that were our contemporaries and at times collaborators during the period from the early 70s to the mid-80s.

## 1. Early History

Steve was born in 1927 in a small town in Hungary, Nagtilav, close to the Austrian border. The location is significant in that when in 1956 Russian tanks approached the town as they crushed the Hungarian uprising, he was able to quickly find sanctuary across the border, eventually reaching the USA in 1957. Prior to this turning point in his life, he had earned a chemical engineering degree in 1950 at the technical university in Budapest. He then held successive positions at two research institutes of the Hungarian Academy of Sciences in microbiology and soil science.

On arrival in the USA, under the auspices of the American Academy of Sciences, he attended 6-week orientation and language courses. He then was admitted to Georgetown University, where he earned a Ph.D. in pharmacology. He never lost his connection to his home country, and in later years established a number of scientific collaborations with Hungarian scientists, and in turn was honored for his contributions, receiving an honorary degree from the University of Debrecen and membership in the Hungarian Academy of Sciences.

## 2. Our First Meeting

The scene was a meeting room at George Washington University, the occasion being a review of progress aimed at establishing a potential role for adenoviruses in human cancer. The participants, myself included, were government contractors in a program overseen by Bob Huebner. Bob’s approach was to stimulate competition and incorporate the “twinning” findings into his overall strategy. My focus was to purify the T “tumor-specific” and virion antigens for use in serological surveys of human cancer patients. My background in immunology coming from a fellowship at Cal Tech, in the laboratory of Ray Owen, and 2 years at the Wistar Institute in Philadelphia working with several DNA tumor viruses [1], was a good foundation for this project. My major “competitor” was Steve’s boss at GWU, an individual whose name I have forgotten. I presented first, confident in my findings, and thus was stunned when these were contradicted in an antagonistic and garbled presentation by Steve’s boss. In the midst of this, suddenly from the back of the room, a slight man jumped from his seat and raced to the front of the room, snatching the chalk from the presenter’s hand. He repeated loudly “No! No! No!” several times and proceeded to quickly outline his work in her laboratory that was in perfect agreement with mine. What had just happened and who was this guy? Whoever this was, it was clear to everyone in the room that he had just lost his job.

The meeting quickly adjourned, we went outside, and I called him over to a quiet place where we could talk. He was understandably distraught and in fragmented sentences told me that he had wanted to talk to me for some time but was forbidden. He knew that what he had done would put his position in jeopardy and he was concerned for his wife and two children. This situation obviously required a fairly rapid resolution. Fortunately, Dr. Huebner had a wonderful assistant, Harriet, who was a classic earth mother. She huddled with Bob and then called me over for a private meeting. Bob has made his decision: if you want to hire this man for your program at Flow Labs, the funding will be arranged. Thus, a partnership was formed, blending my background in immunology with his in biochemistry. Indeed, we soon published our first paper together in 1970 [2] on the topic mentioned above.

I have told this story many times to younger colleagues to illustrate that sometimes the truth compels action, even at great personal risk. We can all learn from Steve’s actions that day. I have never forgotten his courage and devotion to the basic human qualities of honesty and integrity.

## 3. Farewell to Adenoviruses

We now had the tools in hand to conduct our survey of cancer patients, based on animal models. It is interesting that the origin of this effort was a vaccine screening program, and the observations of tumor induction in newborn hamsters led to a complete refocusing of Bob Huebner’s interests. In fact, he left the NIAID (National Institute of Allergy and Infectious Diseases) and joined the NCI (National Cancer Institute), where he was a leading force in the formation of the Special Virus Cancer Program (SVCP) that benefited many investigators. I mention this to honor his memory, as he generously supported our efforts even if they seemed outside of his direct interests. The results of our survey [3] were negative, and thus we could not justify continuing working with this group of viruses. Fortunately, new horizons were opening up to which we could apply our talents.

Transition to Retroviruses: One of our colleagues was fond of saying that he wanted to publish a paper with the conclusion that “no further work need be done on this topic”. As we were funded for a specific purpose and this was evidently met, we needed a rapid transition. Huebner was clearly influenced by the outstanding work of Wally Rowe and Janet Hartley, members of his laboratory at NIAID. These individuals are noted for their work in bringing the principles of quantitative virology to the study of RNA tumor viruses. Instead of just inoculating animals with tissue extracts and waiting to see what would happen, in vitro tissue culture techniques could be used to obtain accurate virus concentrations and also to grow large amounts for use in immunochemical studies. This was a perfect situation for us, and again with modifications of our contract’s work scope, we were on our way.

We wasted no time in setting about purifying the major group-specific antigen of murine leukemia virus (p30gag) in order to develop specific sensitive assays for use in projected epidemiological surveys [4]. We added radioimmunoassays to our capability to provide more sensitive procedures for such studies [5]. The years from 1970 onward saw an explosion in the number of species from which retroviruses—the common term for this family of viruses now used based on their mode of replication as discovered by Temin and Baltimore—were isolated. These included rats, hamsters, cats, several primate species, and eventually the human T-cell leukemia virus by Bob Gallo’s laboratory. We increased our battery of specific reagents for all these isolates and thus became an informal reference and collaborative lab for many in the field—I will reference just a few of these efforts to illustrate this point [6,7,8,9,10,11,12]. We exploited the strategy of successive immunization with related antigens to develop broadly cross-reactive antibodies [13] useful in a variety of assays, with the ultimate goal of use in human surveys.

We also developed the capability for large-scale virus production essential for detailed structural analysis of virus structural components. We were fortunate to obtain one of the first automated protein sequencers made by Beckman and put it to use in the analysis of p30gag from the murine leukemia virus [14]. The ability to do this is obviously dependent on the purity of the test protein, so this was important in many ways. I will never forget the day that Steve came dancing down the hallway at Flow labs, exclaiming “Ray, Ray, Ray, we have it, Pro-Leu-Arg”, the amino terminal sequence with no ambiguity. From this initial starting point, there was no doubt that Steve would go on to a lot more fine structural analysis, not limited to interviral relationships but including functional analysis as well.

## 4. The Move to FCRDC (FCRF)

Our program at Flow Labs was doing well thanks to Steve and also a number of new colleagues who were sent to work with us by Huebner. These included Masa Hatanaka, Hiromi Okabe, Nobuo Tsuchida, all accomplished molecular biologists; Gary Kelloff, Cedric Long, and a long-term associate from the lab of Murray Gardner, Howard Charman. However, in 1975, several factors led to a decision to leave Flow and look for a new home for our program. Fortunately, I had developed a strong relationship with John Moloney, head of the SVCP, and he paved the way for our move to the Frederick Cancer Research Facility located on the grounds of Ft. Detrick. A portion of this facility had been given over to the NCI in 1972 upon closure of the USA Army effort in biowarfare. We accomplished this with amazing ease, adding a valuable new colleague, Nancy Rice, as part of the move and finding an excellent electron microscopist, Matthew Gonda, already present. Of course, a major factor in the move was secure funding along with the ability to define our research goals in broad terms.

We were progressing nicely when a major trial occurred. The NCI division funding our effort was being reviewed by a board of scientific counselors, and now with much greater visibility, as compared to its startup years, we needed to perform. At the board meeting, I presented the main thrust of our work over the years and gained the interest of Jim Watson, one of the board members. Jim put down the newspaper he was reading and announced that he wanted to see this in person with a peer review group. In addition to my old Flow group, I now had responsibility for several existing programs that I was “remodeling” and a large support effort for government scientists; thankfully, these activities were excluded from the review. The entire effort was now called the Biological Carcinogenesis Program (BCP). I knew that Steve would have to perform, and we worked hard to get him to face the audience and convince them that he knew his stuff. He was a reluctant speaker with speech patterns that led to colleagues asking me how it was that I understood him so well, since our interaction resembled a duet with lots of punctuation. I would reply that we understood each other quite well and this did not bother me at all.

The review group included David Baltimore and several other leading experts in the field. I did my stuff and then it was Steve’s turn. He faced front and performed as if he was doing this his whole life. Full of excitement and enthusiasm, with arms waving and the unusual punctuation, he had the reviewers smiling and nodding in agreement as he convinced them that here was a virtuoso who had complete command of his field. I loved it, in a sense like a proud father. This was a key moment in our relationship, and when several years later, we would be in separate organizations, still at the Frederick facility, I was comfortable that I had not let him down. I do not remember how the rest of the group did, but it must have been OK because we ended up with a very favorable site visit report. A few years later, we passed another challenging review at a time when the entire center was under funding pressure from the NCI administration. This led to a conversion from one large contract to five separate ones and which grouped all of the basic research into one and the large operations and technical support activities into another.

## 5. A Fork in the Road

I became head of the Operations contract, and key aspects of the BCP were incorporated into the Basic Research Program (BRP), which in an organizational sense separated me from my long-term colleagues. Fortunately for me, the BRP was headed by a long-term friend and outstanding scientist, George Vande Woude. Steve and Nancy Rice (well known for her work on the *rel* oncogene [15]), with the support of George, kept me involved in an adjunct role. Steve became director of the Molecular Virology and Carcinogenesis Laboratory in the BCP, but our working relationship barely changed [16,17,18], which in hindsight was quite remarkable and another illustration of Steve’s character. During this entire time, Steve expanded his focus on functional–structural relationships among retroviral proteins that led to the understanding of the key role of the viral protease in virus maturation. In turn, this was a finding that led to another key target for drug development for HIV.

The depth and breadth of Steve’s work from 1983 until his retirement in 1995 are noteworthy, and I cannot do them justice in this brief testimonial. One need only do a Google search under his name to appreciate his many contributions to retrovirology. He was especially active in obtaining support for scientists in his native Hungary, as noted in the receipt of an honorary degree and election to the Hungarian Academy of Sciences. He certainly deserved these honors and more.

## 6. Conclusions

My dear friend and colleague Steve Oroszlan was faithful to the principles of his chosen career in science, a model of honesty and integrity. His accomplishments speak for themselves and certainly are deserving of this Special Issue in his honor. My thanks to Alan Rein for giving me the opportunity to provide this brief testimonial that also allowed me to relive an important part of my life. I close with the defining memory of a Hungarian virtuoso dancing down the hallway at Flow Labs, repeating “Ray, Ray, Ray, we did it, Pro-Leu-Arg.” Yes, Steve, you did it!!
